# Bolt Positioning Detection Based on Improved YOLOv5 for Bridge Structural Health Monitoring

**DOI:** 10.3390/s23010396

**Published:** 2022-12-30

**Authors:** Diyong Wang, Meixia Zhang, Danjie Sheng, Weiming Chen

**Affiliations:** Faculty of Engineering, China University of Geosciences, Wuhan 430074, China

**Keywords:** bolt detection, YOLOv5, bridge structural health monitoring, anchor box, feature fusion

## Abstract

To improve the stability of the bridge structure, we detect bolts in the bridge which cause the symmetry failure of the bridge center. For data acquisition, bolts are small-scale objects under complex background in images, and their feature expression ability is limited. Due to those questions, we propose a new bolt positioning detection based on improved YOLOv5 for bridge structural health monitoring. This paper makes three major contributions. Firstly, according to the calibration anchor boxes of bolts, the size and proportion parameters of the initial anchor boxes are optimized by K-means++ clustering algorithm to solve the initial clustering problem of anchor boxes in object detection. Second, the hypercolumn (HC) technique fuses the low-level global features of the trunk and the high-level local features of three different scales to solve the problem of the inefficient distribution of anchors and insufficient extraction of classification features. In this way, we improve the detection accuracy and speed of bolt detection. Finally, we establish a dataset of bridge bolts through network collection and public datasets, including 1494 images. We compare and verify the new method in the collected bolt dataset. The experimental results show that the precision (P) of the improved YOLOv5x is up to 87.3%, and the average precision (AP) is up to 86.3%, which are 6.5% and 5.9% higher than the original YOLOv5x, respectively.

## 1. Introduction

The sudden loss of symmetry stability of the bridge centerline affects the bridge health’s structure and then causes accidents [1]. As the main components commonly used in bridges, bolts can effectively reduce distortions and fatigue stresses and avoid the failure of bridge health structures [2]. The missing and loose bolts caused by long-term exposure to harsh environments affects the overall structural health of the bridge and further cause accidents [3]. Bolt detection usually adopts manual detection. This method has a large amount of detection, low efficiency, and large errors and requires high energy and the experience of inspection personnel [4]. In this way, it not only consumes a lot of manpower and material resources but also has the possibility of missing bolt detection. Therefore, to maintain bridge structure health, it is necessary to develop a real-time accurate detection method for bridge bolts.

Structure health monitoring based on machine learning shows a good ability to detect defects and damage in engineering structures [5]. As a branch of machine learning, computer vision has become an important way and development direction of structure health monitoring [6]. Some studies have proposed that loose bolt detection based on computer vision is an important research direction in structure health monitoring [7], and bolt loosening causes repeated load and vibration of steel structures in bridges. Computer vision object detection is used to locate bolts and realize real-time damage monitoring of bolts, which plays an important role in ensuring the structure health and stability of bridges, vehicles, and dams [8]. The connection status of bolts has an important impact on the safety and reliability of the whole bridge structure’s health [9], so it is necessary to strengthen the application of intelligent technology to detect bolt status in bridge structure health monitoring.

Object detection based on computer vision is a process in which the computer extracts the color, geometry, corner, texture, and other attribute features of the target objects, quickly locates the relevant information in the image, extracts and analyzes the relevant information, and finally understands the target object. Existing object detection algorithms are divided into traditional object detection methods and object detection methods based on deep learning. Traditional object detection methods obtain the features of detected objects in images by manually designing feature descriptors. This method encodes the features and uses machine learning classifiers for detection [10,11,12]. The selection of different descriptors for detected object features affects the detection accuracy. The generalization ability of traditional object detection methods needs to be improved. Object detection methods based on deep learning are mainly divided into regression/classification-based frameworks and region proposal-based frameworks [13]. The region proposal-based framework usually includes a multi-stage process such as region proposal, region classification, and region position adjustment. The region proposal-based framework has the advantages of high detection accuracy and shared calculation parameters, but it has the disadvantages of long training time and slow detection speed. The regression/classification-based framework directly detects the class probabilities and region coordinates of the objects. The framework can detect the object in one stage. Regression/classification-based framework is fast in detection and good at learning the features of objects. This framework mainly includes YOLO series [14,15,16,17] and SSD [18]. Regression/classification-based framework is more suitable for real-time detection, but the accuracy of small-scale object detection needs to be improved.

YOLO series object detection network is widely used in the object detection of bridge structure health monitoring because of its advantages of high precision and fast speed [19]. Deng J et al. [20] detected concrete cracks through the YOLOv2 object detector, effectively assessing the robustness of bridge structures. At the same time, the proposed YOLOv2 detector was compared with Faster-RCNN, and the YOLOv2 detector showed better results in terms of both accuracy and detection speed. Teng S et al. [21] used the improved YOLOv3 to detect surface defects (cracks and exposed rebar) in bridge structure health monitoring. The results showed that the improved YOLOv3 performed best in terms of detection accuracy and speed. Han Q et al. [22] proposed to use the YOLOV3 for panoramic crack location monitoring in view of the structural health monitoring of common steel structure connections, and YOLO V3 proved good performance in both running speed and small-scale object detection. At present, bridge structural health monitoring is mostly based on object detection of cracks in concrete structures. Hou R et al. [23] innovatively proposed the use of YOLO networks to locate trucks and detect trucks in real time to trigger the work of a bridge structural health monitoring system. Bolts are related to the nature of the bridge structure and are one of the key points of bridge structure health monitoring [24]. The bridge structure health monitoring system triggered by real-time bolt positioning detection needs further research.

Bolts on the real scene are small-scale objects in computer vision object detection. The object detection combined with the geometry and texture features of the bolt have become the current research direction. In terms of feature description and classifier training, Ramana L et al. [25] used histograms of oriented gradient (HOG) features and two classifiers to conduct classification training for small-scale bolts. Wang C et al. [26] converted color images into gray images to improve detection accuracy, and they positioned bolts by convolutional neural network digit recognition and detected bolt rotation angles by Hough transform line detection. Dou Y et al. [27] used the template matching method and geometric structure constraints to locate railway bolts. Park J et al. [28] combined Canny edge detector, Hough transform (HT), and circular Hough transform (CHT) to detect nuts. In the region proposal-based framework, Huynh TC et al. [29] firstly converted the color image to the gray level and then generated candidate areas based on a regional convolutional neural network (RCNN) to detect and locate bolts, which enhanced the accuracy of bolt detection. For the real-time positioning detection of bolts, Sun Y et al. [30] marked bolts and trained YOLOv5 to locate and detect the marking circle on bolts. Although the detection accuracy is very high, manual labeling of each bolt is required. Zhao K et al. [31] proposed an improved YOLOv3 algorithm which realizes high-precision detection of bolts through data enhancement, improved clustering algorithms, and fusion features.

Existing object detection methods based on computer vision realize bolt detection through the extraction or constraint of bolt geometric features. Although the above methods achieve high accuracy, most of them still need manual intervention. Bolt detection shows good results in regression/classification-based frameworks, which can adapt to current intelligent real-time detection requirements, but they still lack a high-precision positioning method for bridge bolts.

In this paper, the regression/classification-based framework of YOLOv5 is used to detect bolts on a bridge. The main difficulty of this study is that bolts, as a small-scale objects, make it difficult to extract features and to distribute anchors. It results in insufficient detection accuracy and waste of computing power. The contributions of this study are as follows:

(1) According to the calibration anchor boxes of bridge bolt images, we propose a method based on the K-means++ clustering algorithm to optimize the initial anchor box size and proportion parameters. This method solves the initial anchor boxes clustering problem in new datasets.

(2) To solve the problem that inefficient distribution of anchors and insufficient extraction of classification features, we use the hypercolumn technique to combine the low-level global features of the trunk with the high-level local features of three different scales and use the stairstep upsample structure to generate a single-scale output. This method enhances the detection accuracy and speeds up the detection.

## 2. Improved YOLOv5 Network

**Figure 1:**
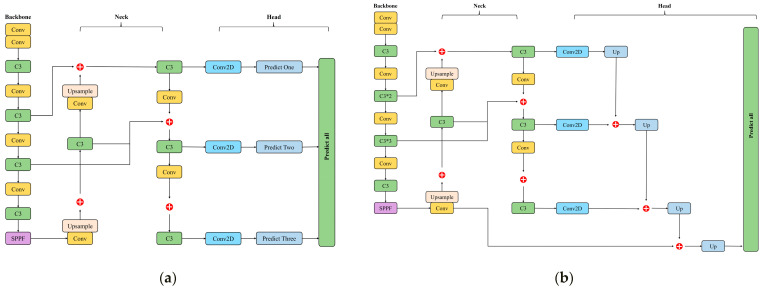
A comparison of YOLOv5x and improved YOLOv5x architecture. (**a**) YOLOv5x; (**b**) improved YOLOv5x.

There are four versions of the YOLOv5 network framework, namely YOLOv5s, YOLOv5m, YOLOv5l, and YOLOv5x. The network depths and widths of the four versions are different. YOLOv5s is the fastest and smallest network version, but its detection performance is also the lowest. YOLOv5x is the network version with the best detection performance, the largest depth and width, and the slowest detection speed. In order to achieve efficient detection of bolts on the bridge, we proposed an improved YOLOv5x. This method first optimizes the anchor box parameters through the K-Means ++ clustering algorithm to improve the accuracy of object detection and location and then creates a single output prediction structure to aggregate the detection feature expression of various scales. The entire architecture is shown in Figure 1.

As shown in Figure 1, the architecture difference between YOLOv5 and the improved YOLOv5 is the head. The redundant structure of the YOLOv5 head is replaced by a lightweight block with HC, which is upsampled with a stairstep structure. At the same time, the improved head uses a single output instead of three to provide higher resolution and speed.

### 2.1. Optimization of Anchor

YOLOv5x applied K-means clustering algorithm for adaptive anchor boxes, and the initial nine preset anchor box parameters were generated based on the COCO dataset. The nine initial anchor boxes in YOLOv5x are divided into three groups: P3/8 is the initial anchor box in the large scale output layer, P4/16 is the initial anchor box in the medium scale output layer, and P5/32 is the initial anchoring box in the small-scale output layer. YOLOv5x sets the maximum threshold (thr) of the width/height ratio of the anchor boxes in the dataset to 4.0. During the training, YOLOv5x clustered the labeled anchor boxes by K-means clustering algorithm under the thr = 4.0 and updated the anchor boxes for the new dataset. K-means clustering algorithm is an unsupervised algorithm that can cluster data into nine specified categories. Before the clustering, nine cluster centers are randomly initialized. Therefore, the clustering method largely depends on the initial selection of the clustering center. In this study, the K-means++ clustering algorithm was adopted to cluster the labeled anchor boxes. It can modify the initial value of the anchor boxes and the thr of the aspect ratio of the labeled anchor boxes. It can improve the accuracy and effect of detection to a certain extent.

**Figure 2:**
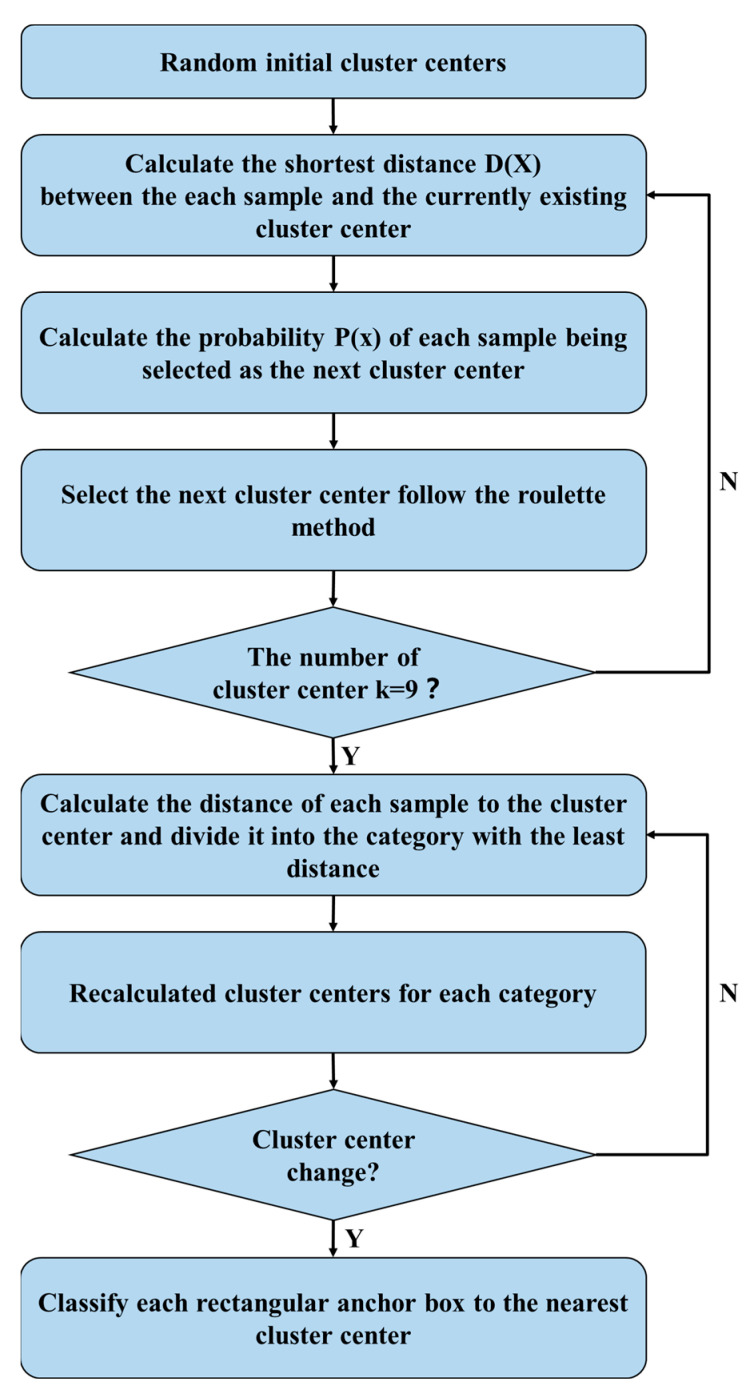
K-Means++ clustering algorithm.

K-means clustering algorithm is an unsupervised learning classification algorithm that can divide a dataset into K categories. The algorithm takes k points as clustering centers to classify the data, and iteratively updates the values of each clustering center to obtain the optimal classification. The K-mean clustering algorithm randomly selects the initial clustering center points, and different selections of the center points affect the clustering results. The K-means++ clustering algorithm is an improvement of the K-means clustering algorithm. When initializing the center point, the clustering centers of different classes are as far away as possible. This improvement effectively avoids the problem that the random selection of the center point is limited to finding the local optimal. The steps of the K-Means ++ clustering algorithm are shown in Figure 2.

**Figure 3:**
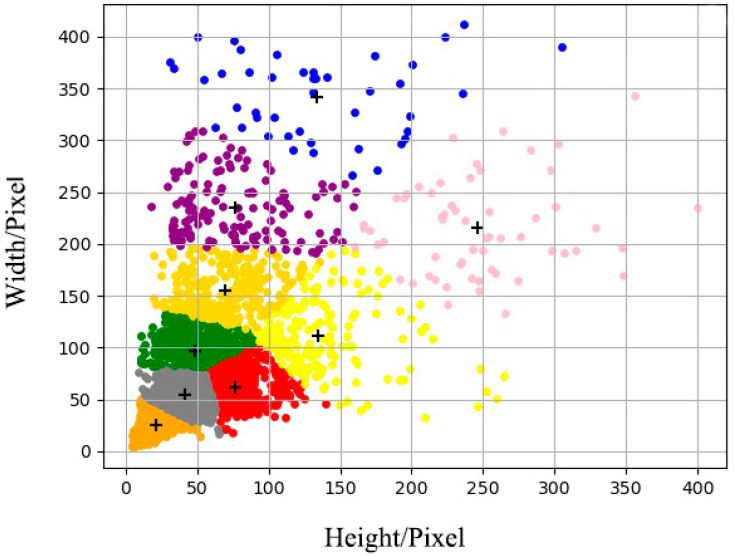
K-means++ algorithm clustering results.

The dataset includes the public dataset and network image collection. In order to obtain the anchor boxes suitable for the dataset, this study set the clustering center k as 9 for the clustering test. The clustering results are shown in Figure 3.

### 2.2. Multi-Scale Feature Fusion

**Figure 4:**
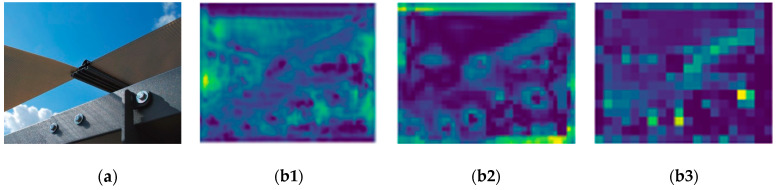
Visual feature maps of three output layers: (**a**) original image; (**b1**) 19*19 feature map; (**b2**) 38*38 feature map; (**b3**) 76*76 feature map.

YOLOv5x trains based on the anchor boxes and detects bolts in three output layers. The initial nine anchor boxes are split into triplets and connected with the large, medium, and small anchor boxes detected in the output layers. The detection object is evenly distributed in large, medium, and small scales, and it can show a good detection ability. However, in the detection process of small-scale objects such as bolts, the object expression ability is weak. The small-scale object feature map input into an improper scale output layer leads to inefficient allocation of anchors and waste of computing power. The small-scale object is mistakenly input into different scale output layers. It not only results in low precision detection but also greatly reduces the use efficiency of the other two output layers. An example of output layer feature map visualization for three scales is shown in Figure 4.

In the feature extraction process of the convolutional neural network, with the increase in network layers, more semantic information of high-level feature description objects is obtained. The YOLOv5 network framework uses upsample to reduce the loss of global features in the network layer number to a certain extent. In the prediction stage, the direct use of the non-maximum suppression (NMS) method is not good for the expression of small-scale object detection. On the actual scene, bolts belong to small-scale objects. The fusion of high-level features and low-level features can express the object features more effectively and improve the detection accuracy.

**Figure 5:**
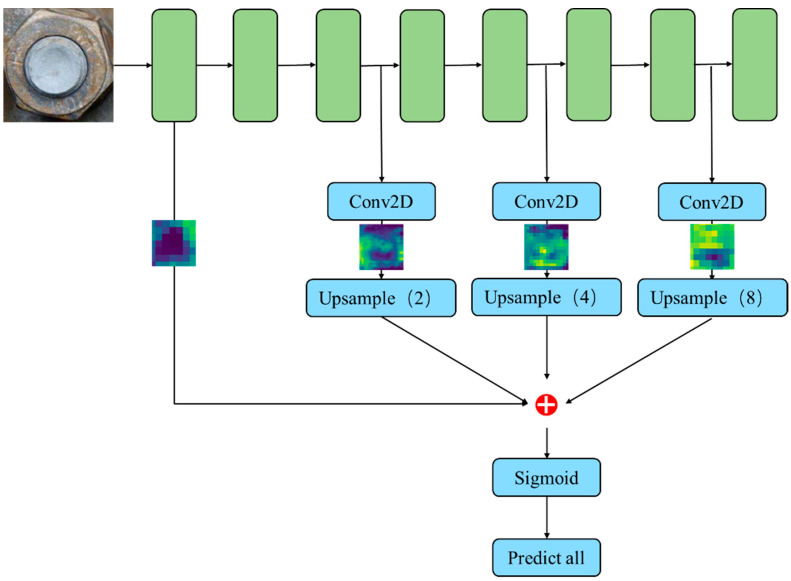
The overall structure based on HC classifiers.

In order to solve the above problems, we propose a network structure with a single output layer to integrate multiple scales. HyperColumn(HC) [32] is a technique that combines the activation values of convolutional network units of all corresponding positions behind an input pixel position into a column vector. The HC classifier takes an anchor box and resizes it to the fixed input size of the corresponding network. In this way, the different scale feature maps are extracted. The bilinear interpolation method is used to resize the feature map, and then the resized feature map is stitched together to obtain a matrix. Each vector in the matrix fuses all the information from the pixel. Finally, sigmiod was used to classify the feature data and obtain the corresponding object prediction result for each pixel. Considering that the bolt is a small-scale object and the background information can enhance the detection accuracy of small-scale objects, we use the fusion method of low-level global features and high-level local features to enhance the detection accuracy. The overall structure based on HC classifiers is shown in Figure 5.

**Figure 6:**
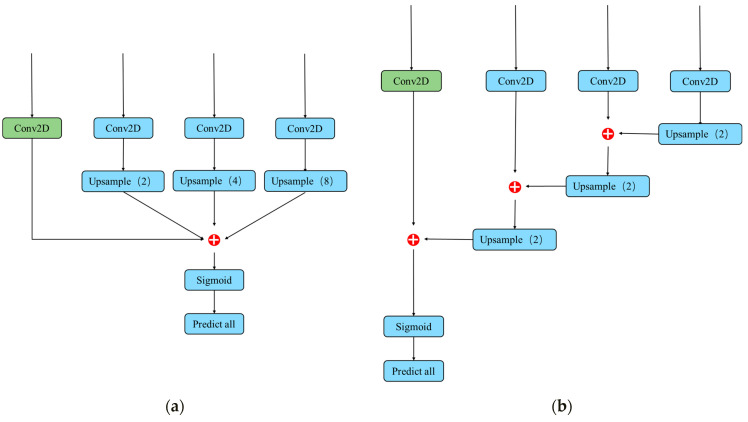
Structure of HC classifier and HC classifier with stairsteps. (**a**) HC classifier; (**b**) HC classifier with stairsteps.

In terms of formula calculation, the output feature map using the HC is shown in Equation (1).

(1) $$F=g\left[F_{1},k\left(F_{2},2^{1}\right),k\left(F_{3},2^{2}\right),\cdots ,k\left(F_{n},2^{n-1}\right)\right]$$

where *F* is the feature map obtained by fusion, *F_n_* is the feature map on different scales, *k*[·, 2*^n^*^−1^] is a function that scales the feature graph to a uniform size by bilinear interpolation, 2*^n^*^−1^ is a scaling factor, and *g*(·) is the function that splices the feature maps. It can be seen from Equation (1) that the existence of scaling factors leads to imbalance in the fusion process. Therefore, we propose a new HC classifier structure with stairsteps. We add the features maps of the previous scale into the fusion process of each step so as to avoid the unbalanced fusion of features in the process. The comparison of the two HC classifier structures is shown in Figure 6.

In terms of formula calculation, the new HC with stairsteps is shown in Equation (2).

(2) $$F^{\prime }=\cdots k\left\{\left[k\left(F_{n},2\right)+F_{n-1}\right],2\right\}\cdots$$

where $F^{\prime }$ is the new feature map obtained by HC with stairstep. It not only solves the problem of inefficient distribution of anchors but also solves the problem of insufficient high-level local features of small targets through the fusion of low-level global features.

## 3. Experiment

### 3.1. Dataset

A dataset of 1494 bolt images was constructed, partly from the public dataset NPU-BOLT [33] (337 images) and partly from the web collection (1157 images). In order to better enhance the robustness of network detection, the bolt background in the dataset includes whiteboards and bridges. It also contains multi-angle and multi-resolution images. Then we used the original Mosaic data enhancement method in YOLOv5. It randomly clips and scales the four images and then randomly arranges and stitches them together to form an image. This data enhancement method not only enriches the data set but also adds small-scale objects to improve the training speed of the network. We selected 1045 (70%) images as the training set, 299 (20%) images as the val set, and 150 (10%) images as the test set. In this paper, LabelImg image annotation tool was used to label the position of bolts in the image, and the corresponding txt file was generated for training. When constructing the samples, the length of the captured images is resized to 640 pixels, and the width is adjusted accordingly to maintain the original proportion of the image.

### 3.2. Details

The experimental environment parameters in this paper are as follows: Windows 11 operating system, AMD R7-5800H central processing unit (CPU), Nvidia GeForce RTX 3060LP graphics processor (GPU), pytorch1.10.0+cu113 algorithm framework, and the programming language is Python3.6. 

The training parameters in this paper are as follows: the number of training steps is 100 epochs; the initial learning rate is 0.01; the optimizer select SGD to update parameters; the batch size is 8; and the image size is 640 × 640.

Average precision (*AP*), precision (*P*), and frames per second (*FPS*) are selected as evaluation indexes to evaluate the model. *FPS* is used to evaluate the speed of object detection, which is the number of images detected by the network per second. The shorter the object detection time of an image is, the faster the network detection speed is. That is, the higher the *FPS* value, the better the object detection network can meet the requirements of intelligent industry real-time detection. The specific calculation formulas for *P* and *AP* are as follows:

(3) $$P=\frac{TP}{TP+FP}$$

(4) $$R=\frac{TP}{TP+FN}$$

(5) $$AP=\int_{0}^{1}P\;Rdr$$

where *TP* is the result of correctly identifying the object sample, *FP* is the result of incorrectly identifying the non-object sample as the object, *FN* is the result of incorrectly identifying the non-object sample as the object, and *TN* is the result of correctly identifying the non-object sample as the non-object. *P* is the accuracy rate, *R* is the recall rate, *R* measures the integrity of the test results, and *P* measures the accuracy of the test. *AP* is the area under the *P*-*R* curve. *AP* is an important index to evaluate an object detection network, and the *AP* value is proportional to the classification performance of the object detection network.

### 3.3. Experimental Results

Among the four versions of YOLOv5, the detection accuracy of YOLOv5x is the highest, and the detection speed of YOLOv5s is the fastest. Therefore, we applied the improved method in the experiment of YOLOv5x and YOLOv5s models. The experimental results are shown in Table 1.

As can be seen from Table 1, AP and P of the improved YOLOv5x network reach 86.3% and 87.3%, respectively, and the detection speed is also improved. The two metrics of AP and P score the highest in the improved YOLOv5x, and the FPS score the highest in the improved YOLOv5s. Compared with the original YOLOv5x, AP increased by 6.5% and P increased by 5.9%, which proved the effectiveness of the proposed method in improving the detection accuracy. Compared with the improved YOLOv5s, the improved YOLOv5x has higher detection accuracy and lower detection speed, and the FPS of improved YOLOv5x is 26.46, which meets the requirements for real-time detection. Considering the actual requirements, the improved YOLOv5x in this paper is superior to other models in the case of comprehensive consideration of detection accuracy and speed.

**Figure 7:**
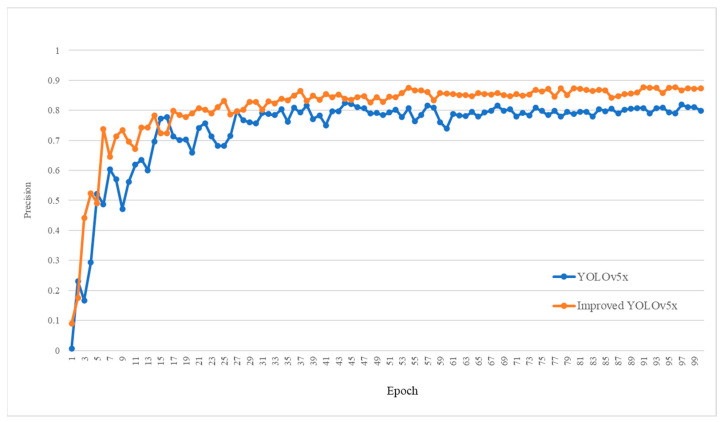
Variation in precision during training epochs.

In order to verify the accuracy and real-time performance of our method, Figure 7 shows the variation in precision during training epochs. A sufficient number of epochs ensures the convergence of the whole training process and enables the module to achieve the best performance under specific parameter configuration. As can be seen from Figure 7, the methods achieve convergence, and the improved YOLOv5 converges after about 30 epochs, while YOLOv5 requires about 60 epochs to reach convergence. Moreover, P of the improved YOLOv5 is always greater than that of the original YOLOv5. Therefore, the new method proposed by us can effectively improve the precision and speed of bolt detection.

In order to more intuitively verify the validity of our method, we selected several representative images and compared the detection results with the original YOLOv5.

**Figure 8:**
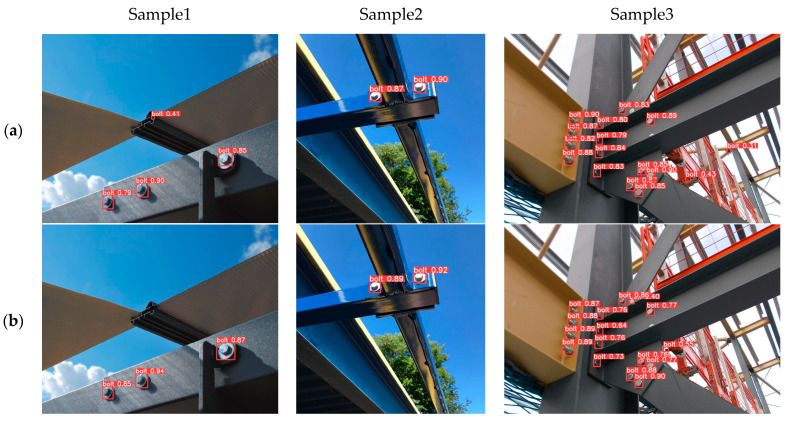
Visual comparison of detection results. The row (**a**): the detection result of YOLOv5x. The row (**b**): the detection result of the improved YOLOv5x.

Figure 8 shows the visual comparison of detection results. The first row (a) is the detection result of YOLOv5x, and the second row (b) is the detection result of the improved YOLOv5x. Compared with sample 1 in the first column, the improved YOLOv5x can effectively avoid false detection. Compared with sample 2 in the second column, the improved YOLOv5x can improve the accuracy of object detection. Compared with sample 3 in the third column, the improved YOLOv5x can effectively avoid false detection and improve detection accuracy in multi-target detection and can detect some distant minimal objects. It can be seen that the K-means++ clustering algorithm and HC with stairsteps can improve the effect of bolt detection. In conclusion, the improved YOLOv5 can improve the positioning detection accuracy of bolts and reduce the misjudgment of small-scale objects, which can verify the effectiveness of the improved measures in this paper.

### 3.4. Ablation Experiments

In order to evaluate the influence of the two improved methods on the results, ablation comparison experiments were conducted on the models. The experimental results are shown in Table 2.

It can be seen from Table 2 that the addition of K-means++ clustering algorithm can effectively improve the detection accuracy for bolts, and the addition of the HC with stairsteps can not only effectively improve the detection accuracy but also improve the detection speed. 

(1) By comparing the experimental results of YOLOv5x and YOLOv5x + Kmeans++, it can be seen that after the improvement of the anchor parameter by the Kmeans++ clustering algorithm, AP and P detected by the model increased by 3.7% and 2.7%, respectively, with no significant change in detection speed. The results showed that the anchor parameter obtained by the Kmeans++ clustering algorithm could improve the accuracy of bolt detection. Therefore, high AP and P values can be guaranteed by selecting appropriate anchor initialization parameters.

(2) By comparing the experimental results of YOLOv5x and YOLOv5x + HC with stairstep, we can see that after the addition of HC with stairstep structures, AP and P detected by the algorithm are improved by 2.9% and 2.1%, respectively, and the detection speed is improved. The results show that the clustering initialization of anchor and the change of network frame parameters can effectively realize the detection of small-scale objects and improve the detection accuracy. HC with stairsteps not only completes the fusion of high-level local features and low-level global features and solves the problem of unbalanced distribution of small-scale object anchor boxes of bolts in the output scale but also avoids the waste of calculation power in the three scale prediction branches. 

(3) Compared with the experimental results of YOLOv5x and improved YOLOv5x, it can be seen that after combining the two improved measures, AP and P detected by the algorithm are improved by 5.9% and 6.5%, respectively, and the detection rate is improved. The results show that the combination of the above two improvement measures can improve the overall detection accuracy of the model, and the detection speed is also improved compared with the original model.

## 4. Conclusions

In this paper, an improved YOLOv5x model is proposed for bolt positioning detection, which is a regression/classification-based framework. Firstly, improved YOLOv5x adopts K-means++ clustering algorithm to learn the proportion and distribution of anchor boxes according to the new dataset. Secondly, the improved YOLOv5x fused the low-level global features of the trunk and the high-level local features of three different scales with an HC technique. In this way, it can solve the problem of the inefficient distribution of anchor boxes and enhance the feature extraction of small-scale objects. Finally, we built a multi-scenario bolts dataset through public datasets and network data acquisition. The improved YOLOv5x was evaluated in the comparison experiment. The results show that our improved YOLOv5x is effective. The final detection AP is 86.3%, P is 87.3%, and an FPS equaling 26.46 was reached, which meets real-time detection requirements.

At the same time, this paper also has some shortcomings. To ensure the bridge structural health monitoring, bolt positioning detection is the first step, and then identifying bolt loosening and wear is the next direction of our research. In order to monitor the health of bridge structures in real time, we will research the relative position judgment of bolts and nuts, calculations of the bolt rotation angle, bolt material judgments, etc.

## Figures and Tables

**Table 1:** Comparison of experimental results.

Model	AP	P	FPS
YOLOv5x	80.4%	80.8%	23.47
**Improved YOLOv5x**	**86.3%**	**87.3%**	26.46
YOLOv5s	76.2%	74.9%	39.37
Improved YOLOv5s	82.6%	82.3%	**53.76**

**Table 2:** Comparison results of ablation experiments.

Model	AP	P	FPS
YOLOv5x	80.4%	80.8%	23.47
**Improved YOLOv5x**	**86.3%**	**87.3%**	**26.46**
YOLOv5x + Kmeans++	84.1%	83.5%	23.87
YOLOv5x+ HC with stairstep	83.3%	82.9%	25.97

## Data Availability

The study used the open data.
